# Exploring Cultural Knowledge in Clinical Work with LGBTQIA+ Patients: A Qualitative Study of Italian Mental Health Professionals

**DOI:** 10.3390/healthcare14142168

**Published:** 2026-07-17

**Authors:** Maria Quintigliano, Gianluca Cruciani, Selene Mezzalira, Cristiano Scandurra, Nicola Carone

**Affiliations:** 1 Department of Systems Medicine, University of Rome Tor Vergata, 00133 Rome, Italy; maria.quintigliano@uniroma2.it (M.Q.); gianluca.cruciani@uniroma2.it (G.C.); nicola.carone@uniroma2.it (N.C.); 2 Department of Human and Social Sciences, Mercatorum University, 00186 Rome, Italy; selene.mezzalira@unimercatorum.it; 3 Department of Humanities, University of Naples “Federico II”, Via Porta di Massa 1, 80133 Naples, Italy

**Keywords:** LGBTQIA+ health, cultural knowledge, cultural competence, cultural humility, mental health professionals, qualitative research, reflexive thematic analysis, minority stress, affirming care

## Abstract

**Highlights:**

**What are the main findings?**

- Italian mental health professionals reported limited and fragmented preparation for working with LGBTQIA+ patients, with formal training often lacking.
- Partial knowledge did not consistently support affirming practice and was often associated with uncertainty in clinical work and difficulty recognizing subtle stigma.

**What are the implications of the main findings?**

- LGBTQIA+ mental health training should be structured, integrated, and practice-oriented, combining knowledge, clinical skills, and supervision.
- Affirming care requires systemic support, including institutional policies and professional standards that make LGBTQIA+ competence a core expectation.

**Abstract:**

**Background/Objectives**: Individuals with marginalized sexual and gender identities experience mental health disparities arising from chronic minority stress and systemic barriers within healthcare settings. Mental health professionals occupy a central role in addressing these inequities; however, persistent deficits in professional preparation and cultural knowledge continue to undermine affirming, identity-inclusive care. This study explored the cultural knowledge of Italian mental health professionals regarding LGBTQIA+ health needs, focusing on how such knowledge is conceptualized, enacted, and constrained in clinical practice, using the Culturally Competent Compassion Model as a theoretical framework. **Methods**: A qualitative study was conducted using semi-structured interviews with 14 Italian mental health professionals (psychologists, psychotherapists, and psychiatrists), purposively selected from a broader qualitative dataset of 33 professionals based on clinical specialization in mental health. Data were analyzed using reflexive thematic analysis. **Results**: Four themes were identified: (1) training as absent structure; (2) knowing without knowing; (3) the paralysis of partial competence; and (4) stigma as institutional climate. Findings showed that professionals often rely on partial and informal knowledge, experience uncertainty in clinical interactions, and encounter difficulties in translating knowledge into practice. Participants demonstrated awareness of explicit and subtle forms of stigma affecting LGBTQIA+ individuals within healthcare settings. **Conclusions**: The findings highlight a gap between declarative awareness and self-reported preparedness for clinical practice, underscoring the need for structured training programs integrating theoretical knowledge and clinical skill development. Cultivating cultural knowledge and cultural humility among mental health professionals represents a systemic investment in reducing mental health inequities and improving affirming care for LGBTQIA+ populations.

## 1. Introduction

Lesbian, gay, bisexual, transgender, queer, intersex, asexual, and other individuals with marginalized sexual and gender identities (LGBTQIA+) face pervasive structural inequalities and interpersonal discrimination that substantially compromise both their psychological well-being and their equitable access to health services [1,2,3,4]. At the interpersonal level, LGBTQIA+ individuals frequently experience microaggressions, stigma, and discrimination in everyday interactions, including within healthcare settings [5,6,7,8,9], while at the intrapersonal level, internalized stigma and expectations of rejection further exacerbate their psychological vulnerability [10,11,12,13]. A substantial body of research has consistently demonstrated that populations with marginalized sexual and gender identities are at increased risk for a range of adverse mental health outcomes, including depression, anxiety, substance use, and suicidal behaviors [14,15]. These disparities are commonly interpreted through the minority stress lens, which posits that chronic exposure to stigma, discrimination, and social marginalization contributes to elevated levels of psychological distress among individuals with marginalized identities [16].

A useful framework for understanding the persistence of health disparities among LGBTQIA+ individuals is the Health Equity Promotion Model [17,18], which conceptualizes health as the result of dynamic interactions among individual, social, and structural factors across the life course. From this perspective, LGBTQIA+ health trajectories are shaped not only by behavioral and psychological processes but also by social positioning, intersecting identities, and broader contextual influences. Structural determinants, such as policies, social norms, and organizational practices, are translated into everyday healthcare experiences through providers’ attitudes, competencies, and clinical decision-making, thereby directly influencing access to care and health outcomes [19]. Discriminatory policies and limited access to inclusive healthcare services further restrict opportunities for equitable care [20,21,22,23,24,25]. Institutional constraints, such as the limited availability of LGBTQIA+-specific services and a shortage of adequately trained practitioners, may substantially hinder access to and utilization of mental health services among LGBTQIA+ individuals [26,27].

Given this convergence of structural, interpersonal, and intrapersonal stressors, mental health services constitute a particularly high-stakes arena for addressing these disparities. However, research indicates that LGBTQIA+ individuals often report lower satisfaction with mental health services than their heterosexual and cisgender counterparts, frequently describing experiences of stigma, lack of understanding, and non-affirming care [28,29,30,31,32]. Psychologists, psychotherapists, and psychiatrists are often required to work with individuals navigating complex identity development processes, experiences of minority stress, and the psychological consequences of stigma and discrimination [33,34,35]. Within this context, clinicians’ knowledge, attitudinal positioning, and competencies in relation to LGBTQIA+ experience play an important role in shaping therapeutic processes and outcomes [1]. Knowledge deficits or attitudinal ambivalence may result in the avoidance of identity-salient material, clinically consequential misreadings of patients’ experiences, or the inadvertent reproduction of heteronormative and cisnormative assumptions that recapitulate, within the therapeutic space, the very dynamics of marginalization from which patients seek relief [36,37,38].

Among the theoretical models developed to conceptualize how healthcare professionals’ competencies and attitudes contribute to individuals’ experience of care, the Culturally Competent Compassion Model [39] provides a comprehensive and process-oriented framework, which has been adapted for LGBTQIA+ populations by Baiocco et al. [40]. This model conceptualizes cultural competence as a dynamic and developmental process encompassing cultural awareness, cultural knowledge, cultural sensitivity, and culturally competent practice. Within this framework, cultural knowledge refers to the acquisition of information regarding the health needs, lived experiences, and social contexts of specific populations. In the case of LGBTQIA+ individuals, this includes knowledge of identity development, minority stress processes, specific mental health vulnerabilities, and the broader social determinants of health.

Alongside cultural competence frameworks, contemporary scholarship in LGBTQIA+ health has increasingly foregrounded the concept of cultural humility. First articulated by Tervalon and Murray-García [41] and subsequently operationalized and empirically examined by Hook and colleagues [42], cultural humility emphasizes ongoing critical self-reflection, an explicit recognition of power asymmetries in the clinical encounter, and a stance of sustained curiosity toward the patient’s self-defined experience rather than reliance on categorical knowledge. Whereas cultural competence frameworks tend to position knowledge and skill acquisition as a progressive developmental achievement toward a definable endpoint, cultural humility reframes professional development as a fundamentally open-ended, relationally constituted, and reflexively sustained orientation.

Empirical research has linked clinician-expressed cultural humility to stronger therapeutic alliances and more positive outcomes across minoritized populations [42], including in LGBTQIA+-specific clinical contexts in which the power dynamics of the therapeutic relationship intersect with broader social marginalization. The cultural competence and cultural humility frameworks are not mutually exclusive; rather, they represent complementary epistemological commitments that together constitute the foundation of affirming practice with LGBTQIA+ populations.

Notwithstanding growing scholarly and policy-level attention to these issues, a substantial body of literature has highlighted persistent gaps in mental health professionals’ knowledge and preparedness in working with LGBTQIA+ populations [1]; professionals often display heterogeneous levels of knowledge and attitudes toward LGBTQIA+ patients, with notable deficits in competence, particularly regarding gender diversity. Moreover, recent guidelines for culturally competent and affirming practices emphasize the need for structured training and the development of specific competencies to improve healthcare provision for LGBTQIA+ populations [3]. Importantly, mental health professionals may possess general awareness of LGBTQIA+ issues while still experiencing uncertainty when addressing these topics in clinical interactions. This knowledge-to-practice gap assumes particular clinical salience in psychotherapy, where practitioners must navigate the intricate interplay of identity, stigma, and relational experience in a manner that is both technically competent and interpersonally affirming [43].

Within this framework, the present study aims to explore mental health professionals’ cultural knowledge regarding LGBTQIA+ health needs, with a specific focus on how such knowledge is conceptualized and applied in clinical practice. Drawing on the Culturally Competent Compassion Model [39,40], this study focuses on the dimension of cultural knowledge and seeks to provide a preliminary understanding of the factors that facilitate or hinder culturally responsive and affirming care for LGBTQIA+ individuals in mental health settings.

## 2. Materials and Methods

### 2.1. Participants

The present study analyzed data from 14 Italian mental health professionals who were interviewed online using a semi-structured format. Participants were purposively selected from a broader qualitative dataset of 33 healthcare professionals based on the following inclusion criteria: (a) professional qualification as a psychologist, psychotherapist, or psychiatrist; (b) active clinical practice in mental health settings; and (c) willingness to discuss experiences with LGBTQIA+ patients. This last selection criterion was established prior to analysis and was not modified in response to emerging findings. While the sample size may appear limited, analysis proceeded until no substantially new thematic content emerged from additional interviews, suggesting adequate informational sufficiency for the study’s exploratory aims. The sample’s sociodemographic and professional information is reported in Table 1.

The study was approved by the Ethical Committee of the University of Naples “Federico II” (protocol no. 10/2024) and the Territorial Ethics Committee Lazio Area 2 (protocol no. 197.24 CET2 utv), and was conducted in accordance with the EU General Data Protection Regulation. Participation in the study was voluntary. All participants provided written informed consent prior to taking part in both the survey and the interview. Participants were informed about the aims of the study, the voluntary nature of participation, and their right to withdraw at any time. All data were treated confidentially, and identifying information was removed from transcripts to ensure anonymity.

### 2.2. Procedures

Participants were recruited as part of a broader online survey targeting healthcare professionals working in Italy, disseminated through professional networks, word-of-mouth recruitment, and social media platforms (e.g., Facebook and Instagram), following a snowball sampling strategy. A total of 173 healthcare professionals completed the survey. At the end of the survey, participants were invited to leave their email address if they were willing to participate in a follow-up interview. Among those who expressed interest, 33 professionals took part in semi-structured interviews.

### 2.3. Data Collection

Data were collected through semi-structured interviews developed by the research team and grounded in the Culturally Competent Compassion Model. The interview guide was designed to explore professionals’ knowledge, perceptions, and experiences related to LGBTQIA+ individuals within clinical contexts. In particular, the questions addressed the following: (1) perceived training needs related to LGBTQIA+ health; (2) knowledge of specific health and psychosocial issues; (3) challenges encountered in clinical practice; and (4) perceptions of stigma, stereotypes, and discrimination within healthcare settings. Interviews were conducted online via Zoom. The interviews lasted approximately 20 min, which is at the shorter end of the range typically associated with reflexive thematic analysis [44,45] and may have constrained the depth and elaboration of participants’ accounts. All interviews were conducted by a female psychology trainee who had received dedicated training in qualitative interviewing techniques prior to data collection. No relationships between the interviewer and participants existed prior to study participation. Throughout the study, the interviewer received ongoing supervision from an experienced member of the research team with expertise in qualitative research and clinical psychology, supporting consistency in data collection and adherence to the study protocol.

All interviews were audio-recorded with participants’ consent and subsequently transcribed verbatim for analysis.

### 2.4. Data Analysis and Trustworthiness

Qualitative data were analyzed using reflexive thematic analysis as outlined by Braun and Clarke [44]. This approach was selected for its epistemological alignment with the study’s constructionist orientation and its explicit recognition of the analyst’s active role in meaning-making. The analysis followed six phases [44]: (1) familiarization with the data; (2) generation of initial codes; (3) development of candidate themes; (4) review and refinement of themes; (5) definition and naming of themes; and (6) selection of representative data extracts. The analytic process, led by the first author, was iterative and involved continuous movement between the data and emerging interpretations. Two members of the research team were involved in coding and thematic development, engaging in regular discussion throughout the analytic process. Particular attention was directed toward identifying both cross-participant patterns and the analytic significance of divergent accounts. No qualitative data analysis software was used; coding and thematic development were conducted manually by the research team.

Trustworthiness was established through multiple strategies: (a) maintaining an analytic journal throughout the coding process; (b) conducting peer-debriefing sessions among team members; (c) systematic discussion of alternative interpretations at each phase of thematic development; and (d) adherence to the COREQ (Consolidated Criteria for Reporting Qualitative Research) guidelines (see Supplementary Materials). Quality was further assessed according to Yardley’s [46] four principles of qualitative research quality: sensitivity to context, commitment and rigor, transparency and coherence, and impact and importance. Consistent with a reflexive paradigm [44], we did not compute inter-coder reliability coefficients, because our aim was not to achieve coding consensus as a marker of quality but to produce a transparent, theoretically coherent interpretation grounded in the data.

### 2.5. Researchers’ Reflexivity

The research team consisted of five academic researchers and clinical psychologists, most of whom had direct professional experience working with LGBTQIA+ individuals in clinical settings. All team members identified as cisgender; while sexual orientations within the group were diverse, none identified as transgender, gender-diverse, or nonbinary. This dual positioning as clinicians and as researchers constituted both an epistemic resource and a site of potential analytic bias. Familiarity with LGBTQIA+ clinical material facilitated a nuanced reading of participants’ accounts and heightened sensitivity to implicit meanings. To guard against the latter, the team engaged in sustained reflexive dialogue to interrogate emergent interpretations, challenge working assumptions, and resist over-identification with participant positions. Divergences in personal and professional standpoints within the team were treated as productive tensions that deepened analytic breadth and guarded against premature interpretive closure. The team’s cisgender composition is itself a relevant reflexive consideration: while this did not preclude rigorous engagement with trans and nonbinary experience, it may have shaped the interpretive horizon in ways that a more gender-diverse team might have navigated differently. This limitation is acknowledged as a structural feature of the study rather than an individual oversight.

## 3. Results

Reflexive thematic analysis generated four themes. Table 2 provides an overview of each theme, its definition, key sub-themes, and illustrative quotes. The themes are presented sequentially below, with each grounded in representative participant extracts and developed through interpretive commentary.

### 3.1. Theme 1: Training as Absent Structure: Professional Formation in an Institutionally Unsupported Domain

Participants consistently identified the absence of formal education on LGBTQIA+ issues as a central limitation in their professional development. This absence reflects a systematic exclusion of marginalized sexual and gender health from the curricula that constitute professional formation in Italian clinical psychology and psychiatry. As such, the gaps participants describe are not individual deficits remediable through personal initiative but outcomes of an institutional architecture that has not positioned LGBTQIA+ competence as a professional baseline.

*“I think that, in general, there should be greater dissemination of knowledge, including basic terminology, because I notice that, particularly among older colleagues rather than younger ones, there is still some confusion”*

(ID 01, 26-year-old cisgender bisexual woman).

This extract points to important dynamics of intergenerational knowledge transmission in the profession. The participant’s observation that older colleagues display more confusion than younger ones suggests that exposure to LGBTQIA+ content has increased in recent professional discourse. The reference to “basic terminology” is telling: the foundational vocabulary of sexual and gender diversity remains nonstandard professional knowledge.

*“Personally, I would focus more on issues related to transgender individuals, as I feel less knowledgeable in this area. I would be interested in attending training courses on affirming care, for example. More broadly, with regard to the LGBTQIA+ population, it would be useful to better understand their fundamental needs, such as those related to coming out and the expression, affirmation, and defense of one’s identity, particularly in terms of when and how this is functional. I am aware, for instance, that there is literature highlighting the impact of a marginalized status; in the case of individuals who are both homosexual and Muslim, it would be interesting to explore how they experience the expression of their identity and gender, and whether this is functional within the cultural context in which they were raised and which they may wish to maintain”*

(ID 03, 31-year-old cisgender gay man).

The second extract demonstrates emergent intersectional awareness, as this participant explicitly situates identity at the confluence of sexual orientation, religion, and cultural belonging. This is notable precisely because it exceeds what formal training would have provided, illustrating a paradox: practitioners develop sophisticated frameworks through informal routes because institutional formation has not supplied them, producing motivated individual learning alongside systemic inequality in professional preparation across the workforce.

### 3.2. Theme 2: Knowing Without Knowing: The Affective and Epistemic Dimensions of Fragmented Knowledge

Most participants described their knowledge of LGBTQIA+ issues as partial, fragmented, and uncertain. Fragmented knowledge is not simply a neutral informational state but carries affective weight: it is experienced as professional inadequacy, generates a subjective sense of clinical precariousness, and shapes practitioners’ engagement with LGBTQIA+ patients in ways that have direct clinical consequences.

*“I believe that those of us who have been working for many years have significant gaps in this area. We tend to rely on information that we have absorbed at a more superficial level, often through general communication. Therefore, there are many aspects we need to better understand in order to respond effectively to patients’ needs. Of course, there are probably many people who are more informed than I am”*

(ID 15, 58-year-old cisgender heterosexual woman).

This extract encodes several distinct dynamics. The phrase “those of us who have been working for many years” signals a temporal dimension of epistemic disadvantage: length of professional experience here operates as an indicator of exposure to an older professional formation that excluded LGBTQIA+ content. The closing self-deprecatory qualification (“there are probably many people who are more informed than I am”) registers the affective residue of this epistemic positioning.

*“I believe that, particularly with regard to gender identity, this is an area that would require further attention, especially when considering transgender individuals, if I had to prioritize. At the same time, it would be important to expand psychotherapeutic knowledge and approaches toward individuals who identify as asexual or intersex. While, in my experience, working with homosexuality now presents fewer difficulties, asexuality and intersexuality remain areas that are still not well understood or sufficiently recognized within psychotherapeutic practice. For this reason, I think it would be important to deepen knowledge in these areas, particularly regarding the type of approach therapists should adopt when working with individuals with diverse sexual orientations”*

(ID 02, 32-year-old cisgender heterosexual woman).

This second extract maps the topology of knowledge unevenness: the participant’s ranking from homosexuality (now manageable) through transgender experience (requires priority attention) to asexuality and intersex conditions (unrecognized in professional practice) may reflect differences in the visibility of LGBTQIA+ topics within broader public and professional discourse. Issues that have received greater attention in educational, clinical, or societal contexts may be perceived as more familiar, whereas less frequently discussed identities appear to remain associated with greater professional uncertainty.

The convergence of these accounts points to a structural condition rather than an individual shortcoming: cultural knowledge, in this context, is neither systematically cultivated during professional training nor adequately scaffolded within clinical supervision structures, producing a heterogeneous and professionally precarious knowledge base with direct implications for the therapeutic encounter.

### 3.3. Theme 3: The Paralysis of Partial Competence: When Awareness Inhibits Rather than Enables Clinical Action

Participants identified a consistent gap between knowledge possession and knowledge application. Partial knowledge, in combination with heightened awareness of its limits, can produce a specific clinical dynamic: a form of inhibitory over-caution in which the practitioner’s sensitivity to the risk of error paradoxically results in avoidance or attenuated engagement with the very material that requires attention. This dynamic is analytically distinct from ignorance; it is the specific consequence of incomplete professional formation in a domain where the stakes of error have become visible.

*“Training on working with individuals experiencing gender incongruence would be particularly important, especially regarding how to position oneself in relation to these patients. For example, the use of chosen names and pronouns is a crucial aspect. Many psychotherapists are not sufficiently aware of the use of neutral pronouns or inclusive language in therapy, which can be particularly helpful and affirming for nonbinary individuals”*

(ID 06, 32-year-old cisgender heterosexual woman).

This extract operates on a dual register: it is simultaneously a knowledge claim and a relational claim. The participant highlights the clinical significance of language, noting that pronoun use and inclusive terminology communicate acceptance and understanding. The phrasing “how to position oneself in relation to these patients” refers to the relational stance from which clinical work proceeds, suggesting awareness that working with nonbinary individuals requires a willingness to be guided by the patient’s own self-definition.

*“Yes, absolutely. In my opinion, especially with regard to transgender individuals and transition processes, it is essential to understand everything related to change, in terms of both physical and psychological aspects, as well as the broader contexts involved. This includes being able to support individuals throughout these processes of change and adaptation. Similarly, for gay, lesbian, and bisexual individuals, there is an important dimension related to both personal and contextual acceptance. Professionals should be better trained to accompany individuals in navigating their choices and processes of self-acceptance”*

(ID 09, 35-year-old cisgender heterosexual woman).

The verb “accompany” in this extract encodes a relational and processual understanding of clinical work closer to cultural humility’s notion of collaborative, patient-led exploration than to the expertise-delivery model implicit in competence frameworks. This participant does not position the clinician as someone who *knows* what the transgender patient needs but as someone capable of sustained, responsive co-navigation. This suggests that some practitioners have arrived at a cultural humility orientation through clinical experience, while lacking the theoretical vocabulary and institutional scaffolding to consolidate it.

Taken together, these findings reinforce the conceptual distinction between declarative knowledge and procedural competence, and underscore the need for training curricula that prioritize experiential learning, reflective practice, and supervised clinical engagement alongside theoretical instruction.

### 3.4. Theme 4: Stigma as Institutional Climate: From Overt Discrimination to the Microaggressive Ordinary

Stigma toward LGBTQIA+ individuals in healthcare settings constitutes an institutional climate: a diffuse, self-reproducing set of norms, practices, and relational microcultures that shapes the conditions of care. Participants’ accounts demonstrated a capacity to identify this climate at varying registers of visibility, from overt discrimination to the subtler phenomenology of microaggressive interactions.

*“For example, during my postgraduate internship, I witnessed cases where individuals were labeled in a simplistic way, such as ‘this person is clearly gay, that is their problem, end of story,’ without any further exploration, solely on the basis of their sexual orientation”*

(ID 30, 30-year-old cisgender heterosexual woman).

The clinical and institutional context of this account (a postgraduate internship) is significant. The participant witnessed this reductive attribution not at the margins of professional culture but at its core: in the supervised clinical environment designed to socialize practitioners into disciplinary norms. The phrase “end of story” encodes the foreclosure of clinical curiosity and the substitution of categorical attribution for genuine assessment.

*“Yes, absolutely. Unfortunately, we are still faced with the paradox of the continued existence of conversion therapies. Even today, I encounter patients who have previously undergone such treatments. At the same time, there are more subtle and harder-to-recognize phenomena. However, if one has a trained eye, it is possible to identify various forms of microaggressions, such as misgendering or difficulties in understanding and supporting individuals’ processes of affirmation and self-affirmation”*

(ID 31, 36-year-old cisgender gay man).

This extract distinguishes between forms of stigma based on their visibility. Conversion practices are named as overt and legally indefensible; microaggressions, including misgendering and failure to support affirmation processes, are positioned as “harder to recognize,” accessible only to those with a “trained eye.” This conditional phrasing implies that the perception of subtle stigma is itself a competence that must be developed, and that without it, practitioners may participate in its perpetuation while remaining subjectively unaware.

Participants’ capacity to identify these dynamics appeared to vary in relation to their prior engagement with LGBTQIA+ issues, with those reporting greater familiarity seemingly better equipped to recognize subtle forms of stigma. This observation has potential implications for the quality of the therapeutic alliance [47].

Collectively, the four themes converge on a coherent picture: a clinical workforce that is broadly aware of the importance of affirming practice but reports lacking the structured knowledge, applied competence, and institutional support required to enact it consistently. This gap between aspiration and self-reported preparedness reflects the absence of LGBTQIA+-specific training from the professional formation of Italian mental health practitioners.

## 4. Discussion

The present study aimed to explore mental health professionals’ cultural knowledge regarding LGBTQIA+ health needs within the framework of the Culturally Competent Compassion Model [39]. The findings highlight gaps in self-reported knowledge, training, and perceived clinical preparedness, as well as a broader awareness of stigma and discrimination within healthcare settings. Interpreted through the lens of the Health Equity Promotion Model [17,18], these findings illustrate how structural determinants, including the absence of standardized training and limited institutional support, may be translated into everyday clinical encounters through providers’ knowledge limitations and self-reported uncertainty. From this perspective, the knowledge deficits documented in this study are the proximal, clinically legible expression of conditions that may perpetuate healthcare inequities for LGBTQIA+ populations [19,48].

Overall, the results suggest that cultural knowledge among mental health professionals is often perceived as insufficient, fragmented, and not fully integrated into clinical practice. This is consistent with previous research indicating that healthcare providers frequently report limited training on LGBTQIA+ issues and express uncertainty in addressing sexual orientation and gender identity in clinical contexts [49,50,51,52].

Regarding the first theme, the framing of training as absent structure rather than as a simple knowledge deficit reorients interpretation: what participants describe is the foreseeable consequence of curricula that have not positioned LGBTQIA+ competence as a professional requirement. This aligns with existing literature showing that LGBTQIA+ health is often underrepresented in medical and psychological training programs [53,54]. Participants positioned this gap not merely as a content-knowledge deficit but as a structural issue with consequences for professional confidence and perceived self-efficacy.

Concerning the second theme, findings indicate that knowledge deficits extend beyond the absence of information to encompass how knowledge is constructed and applied. The concept of “knowing without knowing” captures a specific epistemic condition: practitioners are aware of the contours and limits of their knowledge in ways that generate affective responses, including anxiety and professional uncertainty, that are themselves clinically consequential. Participants described their understanding of LGBTQIA+ issues as partial and uncertain, particularly regarding transgender identities, suggesting that cultural knowledge is often acquired through informal or experiential pathways [55].

Furthermore, participants reported greater uncertainty regarding less visible or less frequently discussed identities, such as asexuality and intersex conditions, suggesting that knowledge gaps are not uniformly distributed but tend to be more pronounced for identities that receive less attention in both public discourse and professional training. In mental health settings specifically, these knowledge limitations carry particular clinical significance: limited understanding of minority stress processes, the complexities of coming out, queer sexuality, and bi-erasure may hinder therapeutic engagement and compromise the quality of psychological interventions [28,56,57,58]. Moreover, insufficient knowledge of transgender and nonbinary experiences, often rooted in binary frameworks, may lead clinicians to pathologize gender diversity or overlook the specific psychological needs associated with gender-affirming processes [2].

With respect to the third theme, the concept of “the paralysis of partial competence” names a specific clinical dynamic: practitioners whose awareness of LGBTQIA+ issues has developed sufficiently to render them alert to the possibility of error, but whose training has not provided reliable procedural guidance, may adopt avoidant or attenuated clinical strategies. These findings underscore the distinction between knowledge and competence, highlighting the need for training programs that integrate both theoretical understanding and practical skills [36,59]. This pattern is consistent with literature on multicultural counseling, which has documented how low perceived self-efficacy in working with minoritized populations can lead to avoidance and reduced therapeutic depth [49,59].

Some participants described approaching LGBTQIA+ patients “like any other patient,” a stance that, while seemingly inclusive, may reflect what Baker and Beagan [60] have termed “professional neutrality”: a posture that risks reinscribing heteronormative and cisnormative norms and overlooking specific stressors associated with a marginalized status. This finding surfaces a central tension in the Italian context: when culturally competent practices are neither standardized nor institutionally mandated, the provision of affirming care becomes contingent on individual motivation, generating a form of inequity in which LGBTQIA+ patients’ access to quality care depends on individual clinicians’ professional development trajectories.

Regarding the fourth theme, framing stigma as institutional climate shifts the analytical register from individual attitudes to broader institutional and organizational conditions. Participants distinguished between overt discrimination, such as conversion therapy practices, and subtle manifestations including microaggressions and misgendering. This distinction is consistent with literature on microaggressions in healthcare settings [5,6], highlighting how subtle bias can be equally detrimental to therapeutic relationships. The coexistence of overt and implicit stigma points to layered discrimination that demands institutional-level intervention [61,62].

Importantly, these findings illuminate the intersection between cultural knowledge and cultural sensitivity in mental health practice. Participants’ accounts suggest that recognition of stigma is closely linked to the depth of cultural knowledge: professionals with a more nuanced understanding of LGBTQIA+ experiences appeared better equipped to identify subtle forms of discrimination, whereas those with more limited knowledge tended to underestimate such dynamics [47,48].

Taken together, these findings support the relevance of the Culturally Competent Compassion Model [39,40] in understanding mental health professionals’ competencies in working with LGBTQIA+ populations. Although the present study focused specifically on cultural knowledge, the results illuminate important connections across the four components of the model. Participants’ accounts suggest that cultural awareness, meaning the recognition of one’s own assumptions and biases, often precedes and shapes the acquisition of cultural knowledge. At the same time, the reported difficulties in applying knowledge in clinical practice point to a gap between cultural knowledge and culturally competent practice.

Importantly, the pattern documented across all four themes is consistent with both the cultural competence and the cultural humility literature [41,42], which argue that clinician development requires not only information provision but also the cultivation of a reflective, self-critical, and relationally responsive orientation. When read through the lens of the Health Equity Promotion Model [17,18], these findings underscore how individual-level knowledge gaps are embedded within broader conditions that may constrain the translation of affirming intentions into consistent clinical practice [63].

The present findings should be interpreted against the specific structural and sociopolitical conditions of the Italian context, which constitutes a particularly instructive national case for examining how institutional environments shape professional preparedness. Although anti-discrimination provisions exist in certain domains, Italy lacks comprehensive national legislation explicitly protecting individuals from discrimination on the grounds of sexual orientation and gender identity; legislative debates concerning civil partnership recognition, same-sex parenting rights, and legal gender recognition remain chronically contested, reflecting deep sociocultural divisions around sexual and gender diversity [21,64].

Within the healthcare system, LGBTQIA+-specific training is not systematically integrated into university curricula for psychology, psychotherapy, or psychiatry, and the availability of specialized services varies considerably across regions [65,66,67,68,69,70]. Recent qualitative evidence has further documented the challenges faced by transgender individuals in navigating Italian healthcare institutions, highlighting persistent deficits in provider preparedness and structural responsiveness [71]. These conditions do not merely constitute contextual background; they may actively configure the institutional incentives, training priorities, and professional climate within which mental health practitioners develop, or fail to develop, the competencies required for affirming practice. Accordingly, individual-level training initiatives, however necessary, may be insufficient in the absence of coordinated structural reform and enforceable policy frameworks that situate LGBTQIA+-affirming competence as a professional and institutional obligation.

### 4.1. Limitations

Several limitations should be acknowledged. First, the study is based on a relatively small sample of 14 mental health professionals recruited within the Italian context, which may limit generalizability. Second, voluntary participation may have introduced self-selection bias, potentially leading to overrepresentation of professionals already sensitized to LGBTQIA+ issues. Third, the sample was predominantly composed of cisgender women from Central Italy, which may further constrain representativeness. Fourth, the interview duration of approximately 20 min represents a methodological constraint; reflexive thematic analysis is optimally suited to richer, extended qualitative data. The analytic claims advanced should accordingly be understood as exploratory and hypothesis-generating rather than as fully saturated interpretive constructions. Future research should employ longer interview formats.

### 4.2. Future Directions

Future research should address the present study’s limitations through specific methodological improvements. First, longitudinal intervention studies should evaluate the effectiveness of structured LGBTQIA+ training programs using pre-post designs with validated measures of knowledge, attitudes, and clinical self-efficacy [49]. Second, research incorporating LGBTQIA+ patients’ perspectives through parallel qualitative interviews or dyadic analysis would provide complementary evidence on whether provider-reported knowledge gaps translate into patient-perceived care quality. Third, comparative studies across Italian regions and other national contexts would help identify how regional policy variations (e.g., presence of LGBTQIA+ health centers, local anti-discrimination ordinances) shape professional preparedness. Fourth, future studies should recruit larger, more diverse samples including practitioners from Northern Italy, public healthcare settings, and those without prior LGBTQIA+-specific training to test whether the themes identified here replicate across different professional subgroups.

### 4.3. Clinical Implications

The findings of this study have important implications for clinical practice, professional training, and healthcare policy. First, they highlight the need for structured and mandatory training programs on LGBTQIA+ health within both academic curricula and continuing professional education. Such programs should address not only basic knowledge but also specific clinical skills, including the use of inclusive language, the management of identity-related issues, and the understanding of minority stress processes [50,53]. Training curricula should ensure adequate coverage of the full spectrum of sexual and gender diversity, with particular attention to identities that are currently underrepresented in professional education, such as asexuality, intersex conditions, and nonbinary gender identities.

Second, training initiatives should adopt an integrated approach that combines theoretical knowledge with experiential learning, allowing professionals to develop confidence and competence in working with LGBTQIA+ patients. Supervision, reflective practice, and case-based learning may be particularly useful in this regard [59]. Given the knowledge-to-practice gap documented in this study, supervised clinical placements with LGBTQIA+ patient populations may be especially valuable as a structural mechanism for translating declarative knowledge into affirmative clinical competence. Importantly, such training should be explicitly framed within a cultural humility orientation, foregrounding reflective self-awareness and the relational dimensions of affirming practice rather than positioning clinicians as experts in predefined LGBTQIA+ needs. Such training should also foster clinicians’ awareness of their own personal dispositions, which may shape how they navigate the relational and emotional complexity of clinical work with LGBTQIA+ patients [72].

Third, the findings underscore the importance of adopting an intersectional perspective in both training and clinical practice. Participants’ accounts highlighted the complexity of working with patients whose LGBTQIA+ identities intersect with other dimensions of diversity, such as cultural background, religious affiliation, and migration status. Training programs should therefore equip professionals to recognize and address the ways in which multiple, intersecting forms of marginalization shape patients’ experiences and mental health needs [73,74].

Fourth, the findings suggest that efforts to improve LGBTQIA+ healthcare should not be limited to individual competencies but should also address institutional and cultural factors. Promoting inclusive healthcare environments, developing organizational policies that explicitly affirm LGBTQIA+ identities, and reducing stigma within professional settings are essential steps toward improving the quality of care [55]. At the policy level, the integration of LGBTQIA+-specific content into national accreditation standards for psychology, psychotherapy, and psychiatry training programs would represent a structural intervention with the potential to shift the prevailing paradigm in which affirming competence is contingent on individual motivation, toward one in which it constitutes a professional baseline expectation and a condition for institutional accreditation.

## 5. Conclusions

This qualitative study explored the cultural knowledge of Italian mental health professionals regarding LGBTQIA+ health needs, identifying four themes: training as absent structure, knowing without knowing, the paralysis of partial competence, and stigma as institutional climate. Participants demonstrated awareness of the importance of affirming practice while reporting that their knowledge was incomplete and difficult to apply clinically. These findings underscore the need for structured, competency-based training programs that integrate theoretical knowledge with supervised clinical practice, grounded in principles of cultural humility. Moving from informal, self-directed learning toward systematic educational frameworks represents a necessary step toward reducing mental health inequities and ensuring affirming care for LGBTQIA+ populations.

## Figures and Tables

**Table 1:** Sociodemographic and professional information of participants (*N* = 14).

Variables	Sample (*n* = 14)
**Sociodemographic variables**	**M ± SD or *n* (%)**
*Age* (years)	38.6 ± 10
*Gender*	
Cisgender male	2 (14.29)
Cisgender female	12 (85.71)
*Sexual orientation*	
Heterosexual	9 (64.29)
Gay/Lesbian	2 (14.29)
Bisexual	2 (14.29)
Other	1 (7.14)
*Ethnicity*	
White (Caucasian, European)	13 (92.86)
Hispanic or Latino	1 (7.14)
*Marital status*	
Single	5 (35.71)
In a relationship	6 (42.86)
Married	3 (21.43)
*Number of children*	
0	11 (78.57)
1	3 (21.43)
*Geographical location*	
Central Italy	12 (85.71)
Southern Italy and the Islands	2 (14.29)
*Educational level*	
University degree	3 (21.43)
PhD or equivalent	11 (78.57)
*Membership in LGBTQIA+ associations*	
Yes	1 (7.14)
No	13 (92.86)
**Professional activity variables**	***n* (%)**
*Professional experience*	
1–5 years	3 (21.43)
5–10 years	1 (7.14)
10–20 years	3 (21.43)
>20 years	7 (50.00)
*Employment type*	
Full-time	11 (78.57)
Part-time	3 (21.43)
*Employment sector*	
Private sector	10 (71.43)
Both private and public sectors	4 (28.57)
*Treated LGBTQIA+ patients*	
Yes, more than 15	2 (14.29)
Yes, between 6 and 15	2 (14.29)
Yes, between 1 and 5	7 (50.00)
No	3 (21.43)
*Training on specific LGBTQIA+ issues and needs*	
Yes, they were useful	9 (64.29)
No, but I consider it a useful opportunity	5 (35.71)

Notes. M = Mean; SD = Standard Deviation; *n* = number.

**Table 2:** Overview of themes, definitions, and illustrative quotes.

Theme	Definition	Key Sub-Themes	Illustrative Quote
1. Training as absent structure	Systematic exclusion of LGBTQIA+ health from professional curricula, producing knowledge gaps that are structurally rather than individually determined	Absence of formal education; intergenerational knowledge transmission gaps	*“I think that, in general, there should be greater dissemination of knowledge, including basic terminology, because I notice that, particularly among older colleagues rather than younger ones, there is still some confusion”* (ID 01)
2. Knowing without knowing	Partial knowledge that carries affective weight, experienced as professional inadequacy, generating uncertainty that shapes clinical engagement	Fragmented and uncertain knowledge; awareness of one’s own knowledge limits; professional self-evaluation; cohort effects in knowledge distribution	*“I believe that those of us who have been working for many years have significant gaps in this area. We tend to rely on information that we have absorbed at a more superficial level, often through general communication”* (ID 15)
3. The paralysis of partial competence	A clinical dynamic in which partial knowledge combined with awareness of its limits produces inhibitory over-caution and avoidance of identity-salient material	Gap between knowledge and application; inhibitory awareness; need for relational positioning; use of inclusive language	*“Training on working with individuals experiencing gender incongruence would be particularly important, especially regarding how to position oneself in relation to these patients”* (ID 06)
4. Stigma as institutional climate	Stigma as a diffuse, self-reproducing set of institutional norms and practices that shapes conditions of care across multiple registers of visibility	Recognition of overt discrimination; identification of microaggressions; trained perception of subtle bias; conversion therapy legacy	*“I witnessed cases where individuals were labeled in a simplistic way, such as ‘this person is clearly gay, that is their problem, end of story,’ without any further exploration”* (ID 30)

## Data Availability

The data presented in this study are available on request from the corresponding author. The data are not publicly available due to privacy reasons.

## Supplementary Materials

The following supporting information can be downloaded at https://www.mdpi.com/article/10.3390/healthcare14142168/s1: Table S1: COREQ—Page Numbers.

## Abbreviations

The following abbreviation is used in this manuscript:

LGBTQIA+	Lesbian, gay, bisexual, transgender, queer, intersex, asexual, and other marginalized sexual and gender identities
