# Supplementary material for: Exploring Cultural Knowledge in Clinical Work with LGBTQIA+ Patients: A Qualitative Study of Italian Mental Health Professionals
Source: Healthcare (Basel). 2026 Jul 17;14(14):2168. doi: 10.3390/healthcare14142168 (PMC13409998; doi:10.3390/healthcare14142168)
Supplement: Supplementary file 1 [file healthcare-14-02168-s001.zip › healthcare-4255820-supplementary.pdf]

Supplementary Materials

Table S1. COREQ—Page Numbers

Item	Page(s)
1. Interviewer/facilitator	p. 6
2. Credentials	p. 6
3. Occupation	p. 6
4. Gender	p. 6
5. Experience and training	p. 6
6. Relationship established	pp. 7
7. Participant knowledge of interviewer	p. 7
8. Interviewer characteristics	pp. 7-8 (Researchers' Reflexivity)
9. Methodological orientation and theory	p. 7 (Reflexive Thematic Analysis)
10. Sampling	pp. 5-7
11. Method of approach	p. 7
12. Sample size	p. 5
13. Non-participation	Not reported
14. Setting of data collection	p. 6-7
15. Presence of non-participants	Not reported
16. Description of sample	pp. 4-6 (Table 1)
17. Interview guide	p. 6
18. Repeat interviews	Not reported (No)
19. Audio recording	p. 7
20. Field notes	Not reported (No)
21. Duration	p. 6
22. Data saturation	p. 4 ("informational sufficiency")
23. Transcripts returned	Not reported (No)
24. Number of data coders	p. 7
25. Description of coding tree	pp. 8-9 (Table 2)
26. Derivation of themes	p. 7
27. Software	p. 7
28. Participant checking	Not reported (No)
29. Quotations presented	pp. 8-13
30. Data and findings consistent	Results section (pp. 8-13)
31. Clarity of major themes	pp. 8-13
32. Clarity of minor themes	pp. 8-13
